# Breast cancer clustering integrating complete gene expression profiles and genetic ancestry

**DOI:** 10.1371/journal.pone.0352514

**Published:** 2026-07-24

**Authors:** Johanna Stepanian, Alejandro Mejia-Garcia, Carlos Orozco, Jorge Duitama

**Affiliations:** 1 Systems and Computing Engineering Department, Universidad de los Andes, Bogotá, Colombia; 2 Human Genetics Department, McGill University, Montreal, Canada; 3 Instituto Nacional de Cancerología, Bogotá, Colombia; OMICS, PERU

## Abstract

Breast cancer (BC) remains the leading cause of cancer-related mortality among women globally. Precise subtyping of BC is critical for optimizing treatment strategies. This study explored the capacity of bulk RNA-seq data to improve breast cancer characterization by analysis of complete expression profiles. We analyzed RNA-seq data for 274 tumor samples and six healthy tissue samples from diverse geographical origins. Using over 9,800 SNPs directly genotyped from RNA-seq data, we successfully predicted broad genetic ancestry, identifying European, African, Asian, South Asian, and Admixed American origins. Molecular subtyping through PAM50 showed some level of ambiguity, depending on the amount of samples provided as input. *In silico* drug sensitivity analysis identified potential therapeutic strategies, including Etoposide and Mistaurin, with cluster-specific efficacy. Our findings emphasize the integration of ancestry-informed data and complete transcriptomic profiles to redefine BC subtyping. These insights offer a foundation for more equitable, ancestry-informed therapeutic strategies and highlight the importance of diversity in cancer research.

## Introduction

Breast cancer (BC) is the first leading cause of death in women around the world [1]. Subtypes are important to determine the optimal treatment plan for patients [2]. Classic subtypes are determined by the activity of the estrogen receptor (ER), the progesterone receptor (PR), and the human epidermal growth factor 2 (HER2), measured by immunohistochemistry (IHC) [3]. Based on these biomarkers, the most aggressive subtype is the triple-negative BC (TNBC), which is negative for ER, PR, and HER2 [4,5]. Cytotoxic chemotherapy is the main effective therapeutic modality for this subtype. However, most patients present side effects including infertility, osteopenia, and heart damage and some patients develop resistance to the treatment [6].

Despite the availability of high throughput gene expression measurements such as RNA-seq, subtyping is currently performed using the IHC classification method, based on microarray data. This method defines five intrinsic subtypes: Luminal A, Luminal B, Normal-like, Basal and enriched-HER2 [7]. Luminal A shows a good prognosis, a low relapse rate, higher survival time, and sensitivity to endocrine therapy [8–11]. It is usually associated with somatic mutations in *PIK3CA*, *GATA3*, and *MAP3K1* genes, and with overexpression of the cyclin D1 gene [12]. Luminal B presents a lower sensibility to endocrine treatment and a higher sensitivity to chemotherapy, compared to Luminal A [13,14]. It also shows the worst prognosis within the Luminal subtypes [15]. Many patients with germline mutations in *BRCA1* develop basal tumors [16–18]. These tumors are highly diverse in terms of epidemiological, phenotypic, and molecular characteristics, with different patterns in terms of relapse [19,20]. Enriched-HER2 tumors show high expression of genes associated with cellular proliferation [10]. They also show intermediate expression in luminal genes (*ESR1* y *PGR),* and low expression of basal genes and proteins [21]. These tumors have a good response to monoclonal antibody therapy with Trastuzumab, decreasing the death rate in early metastatic states [10]. Resistance to the treatment has been related to overexpression in CXCR4 and the loss of PTEN [22,23]. Finally, normal-like tumors have a different expression pattern and the worst prognosis for the patient [22].

It is known that the most aggressive intrinsic subtypes are more frequent in Latinas, Native American, and African American women compared to European descent women [24]. Although specific factors explaining a higher incidence of HER2 + tumors in Latinas are unknown, a positive correlation between the proportion of native american ancestry and HER2 status has been reported [25]. Higher rates of HER2 gene expression in the HER2 + subtype have also been reported in southeast Asian patients [26–29]. Additionally, in the United States, these ethnic groups have limited access to health services due to multiple cultural and language barriers [30]. While socioeconomic factors contribute to population-based differences in mortality, they do not explain differences in all populations [31].

While immunohistochemistry (IHC) remains widely used due to its cost-effectiveness, it fails to fully capture tumor heterogeneity [32]. Hence, different commercial kits based on gene expression for BC subtyping were developed and are used in clinical practice. One of the most popular methods, known as PAM50, performs supervised clustering of the expression data obtained from a microarray of 50 genes [33–36]. The PAM50 model uses nearest-centroid classification for molecular subtype assignment [35]. These centroids are used to classify new samples [37]. The PAM50 model is FDA approved for prognosis [38], However, it has been questioned if the PAM50 subtypes are clinically and molecularly relevant, due to the limited evidence for chemotherapy decisions and the lack of diversity included in clinical trials [39]. Other panels have been designed such as the Oncotype DX assay, which based on the expression patterns of 21 genes classifies patients into three groups based on the recurrence risk: high, intermediate, and low [40–42].

A main limitation of the current classification models is that they have been trained using data primarily from European populations. Hence, they overlook the heterogeneity of breast cancer in patients with diverse genetic ancestries [31,43,44]. Van Alsten and collaborators reported an over estimation of bad prognosis among African descendant populations [45]. Other studies have shown racial differences in PAM50 subtype distribution [44,46]. Differences in tumor biology and subtype distribution across African, Latin American, and Asian populations have been reported, emphasizing the need for a more inclusive approach to molecular classification [30,31,47–51]. Novel methods including a broader set of genes, more balanced training databases, and ancestry-informed markers are needed to improve diagnostic precision and to achieve equitable, personalized treatment strategies for all breast cancer patients.

In this study, we aim to explore the capacity of bulk RNA-seq data to improve breast cancer characterization by analysis of the nearly complete gene expression profiles that can be reconstructed from RNA-seq data. First, we validated that accurate ancestry predictions can be obtained from direct analysis of RNA-seq data. A clustering of 204 human BC tumors using available RNA-seq data for 475 selected genes provides new groups with improved relationships with cell populations and predicted responses to drug treatments.

## Results

### Genetic ancestry prediction for breast cancer samples from RNA-seq data

We analyzed RNA-seq data from tumors of 274 breast cancer patients and six healthy tissue controls, retrieved from 36 publicly available studies. Most of these studies were conducted in the United States and Spain (S1 File). Reads from all samples included in this study were aligned to the reference genome with a mapping rate exceeding 70%. To assess whether the genetic ancestry of each sample could be evaluated from RNA-seq data alone, we genotyped known variants in coding regions from the 1,000 Genomes Project, which included 1,600 reference female individuals in their project. This resulted in 9,578 single nucleotide variants (SNVs) genotyped in a total of 1,808 individuals with a missing data rate of 1.85%, attributed to RNA-seq differences in expression. The number of genotyped SNVs ranged between 4524 (47.23%) to 9578 (100%), with an average percentage of 88.18% (S1 File). Only 44 samples had a percentage of genotyped sites below 75%.

**Fig 1:**
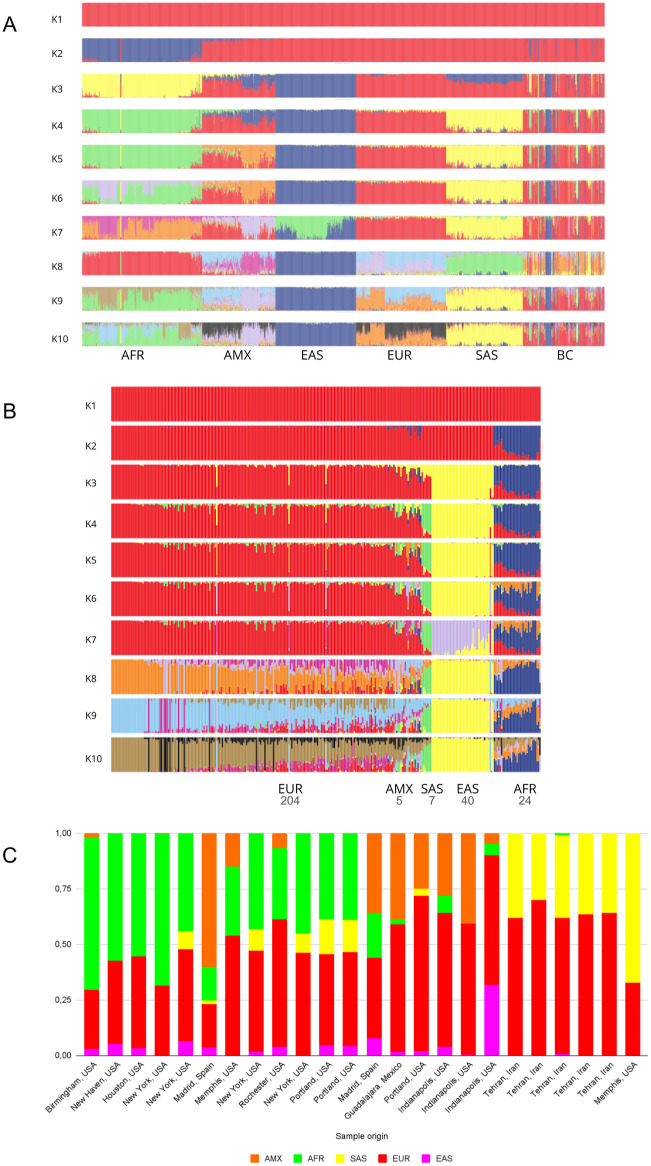
Genetic ancestry. A. ADMIXTURE clustering of individuals from the 1,000 genomes project and breast cancer RNA-seq data. African (AFR), Admixed American (AMX), East Asian (EAS), European (EUR), and South Asian (SAS). B. Ancestry frequency for 280 breast tissue samples by origin. C. Ancestry prediction for the admixed individuals, each column represents an individual.

To infer the genetic population of origin of each sample, we performed a maximum likelihood estimation of individual ancestries using the previously called SNVs varying the number of clusters (k parameter) between 1 and 10, using the genomic variation database constructed from RNA-seq data as input. Ancestry was successfully predicted for samples from the 1,000 Genomes Project (Fig 1A). In particular, the African population was split in k = 2 given its higher diversity compared to other populations. Asian and European groups split in k = 4, and the probably Native American component (AMR, orange) appears in Admixed Americans in k = 5, separating them from the EAS population. We chose k = 5 to determine the ancestry of the breast cancer samples, considering the previous annotations on human populations. Most of the samples (n = 204) were classified as European ancestry (EUR), followed by East Asian (EAS) (n = 40), African (AFR) (n = 24), South Asian (SAS) (n = 7), and Admixed American (AMX) (n = 5) (Fig 1A), which is consistent with the geographic origin of the samples. Individuals from the USA, Israel, Canada, Germany, Spain, the UK, and the Netherlands showed predominantly European ancestry, whereas individuals of Asian origin, including samples from China and South Korea, were classified as EAS. Individuals from Singapore and India clustered predominantly within the SAS population (Fig 1B). Genetic admixture was predicted for individuals from New York, individuals of African origin living in Birmingham, and individuals of Mexican origin (Fig 1C). RNA-seq data for five admixed samples (HG01113 and HG01125 from Colombia, NA19648, NA19654, NA19657 from Los Angeles) was reanalyzed to validate the consistency of ancestry estimates obtained from DNA and RNA-seq data (S1 Fig in S6 File).

Besides the five individuals classified as AMX, we observed 24 individuals with admixture patterns. Most of these individuals were originally classified as European. However, they had a membership probability lower than 0.6 to a single population so we re-classified them as: European–African (25%, 6/24), followed by European–South Asian, and European–Admixed Americans with an equal proportion of 21% each (5/24) and European–East Asian (4%, 1/24). Additionally, we found African–Europeans 21% (5/24), South Asian – European (4%, 1/24), Admixed American–African (4%, 1/24).

### Uncertainty in breast cancer subtyping based on the PAM50 panel

Given that the selected samples were collected from 34 different studies, which used different protocols for RNA extraction and library preparation, it was likely that read counts were affected by different patterns of batch effects. A principal component analysis of a subset of 204 samples belonging to the 12 projects with the largest number of samples corroborated this situation (S2 Fig in S6 File). Taking into account that batch correction is more effective if the groups are well represented, and that subtype prediction based on GeneFu also required more than one sample, we restricted the analysis of expression data to these 204 samples. To assess the stability of GeneFu predictions using the PAM50 model, we performed the prediction of each sample with two different inputs: 1) corrected counts for the 204 samples; and 2) uncorrected counts for the samples within each project.

**Fig 2:**
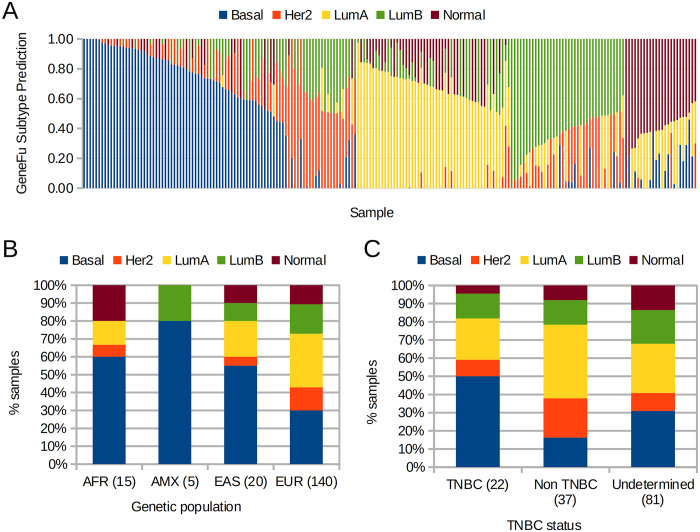
Subtype predictions. A. Subtype frequency obtained with the PAM50 algorithm for 204 RNA-seq breast cancer tumor samples. (LumA = Luminal A, LumB = Luminal B). B. Percentage of 180 samples with consistent subtype predictions discriminated by genetic population. C. Percentage of 140 samples of European origin with consistent subtype predictions discriminated by TNBC status. Numbers in parenthesis correspond to the total number of samples.

Fig 2A shows the distribution of subtype assignment probabilities for each sample if corrected counts for the complete set of samples are taken as input. For 73 samples the maximum probability was lower than 0.6 and for 21 of these samples the probability was lower than 0.5. Consequently, 24 samples (11.76%) had discordant subtyping between the two prediction methods. From the samples with consistent assignments between methods, 66 were classified as Basal, 48 as Luminal A, 26 as Luminal B, 20 as HER2+ and the remaining 20 as Normal like. Within these samples, 10 classified as Luminal A (20.8%) had a probability larger than 0.4 of belonging to another group. A similar situation was observed for 5 samples subtype as Luminal B and 5 samples subtyped as HER2 + .

We investigated the relationship between the subtypes predicted using the PAM50 algorithm to the genetic populations inferred by ADMIXTURE (Fig 2B). Consistent with the distribution of the complete dataset, 140 (77.8%) of the 180 samples with consistent subtype predictions had European ancestry (EUR), while only 5 samples were admixed american (AMX). The group was completed by a nearly even distribution between African (AFR) and east asian (EAS) ancestry. The most noticeable difference in subtyping among ancestry groups was observed for the Basal subtype. While only 30% of the samples with consistent subtyping and European ancestry were subtyped as Basal, the same percentage was higher (>55%) for other population groups, although the difference was not considered significant after correction for multiple testing (p-value > 0.01 for a one sided Fisher exact test). Regarding the correlation between the intrinsic subtype predicted by PAM50 and TNBC status, although more than half of the samples did not have TNBC as reported metadata, we could assess that 47.22% of the 36 individuals cataloged as TNBC had Basal as predicted subtype. This percentage is significantly higher than the 14.29% of the 42 non-TNBC samples having Basal subtype (p-value = 3.50 × 10^−4^ for a one sided Fisher exact test). The difference between percentages increases if only the 140 samples with consistent subtypes and European ancestry are taken into account (Fig 2C, 50% vs 16.22%, p-value = 7.05 × 10^−3^).

### Clustering of breast cancer tumors based on complete expression profiles

**Fig 3:**
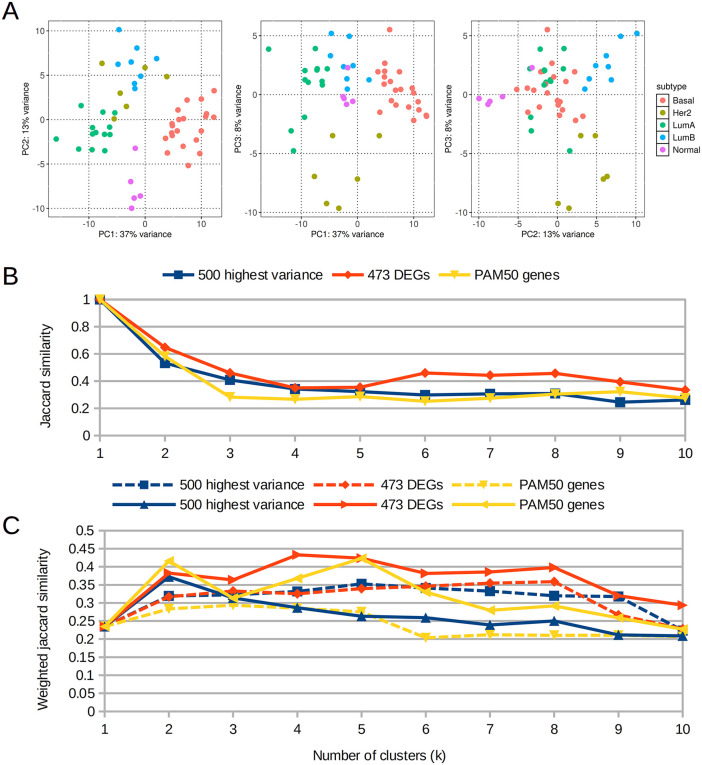
Unsupervised clustering. A. PCA plot of the normalized counts for 476 DEGs selected from 53 RNA-seq samples with consistent subtype assignments. B. Jaccard similarity between clusters obtained by k-means and by hierarchical clustering from different sets of genes expressed in 204 samples. C. Weighted jaccard similarity between clusters obtained from different sets of genes expressed in 204 samples and clusters inferred from predicted subtypes. The jaccard similarity is weighted by the probability of subtype assignment. Continuous lines correspond to k-means and dotted lines correspond to hierarchical clustering.

Considering the potential uncertainty of predictions obtained with the PAM50 algorithm, we investigated analysis alternatives that could lead to a more clear differentiation among subtypes from complete expression profiles. First, we selected a group of 53 samples from the four projects with the largest numbers of samples, having European origin and having consistent subtype predictions (see methods for details). A set of 475 differentially expressed genes (DEGs) were selected performing the 10 possible pairwise comparisons between each pair of subtypes and taking the union of DEGs obtained in each comparison (S2 File). A principal component analysis (PCA) of the corrected counts within these genes visually places samples from different subtypes in consistent regions of the space (Fig 3A). The first principal component explains 37% of the variation and differentiates basal samples from most non-basal samples. The second component explains 13% of the variance and seems to differentiate Normal-like samples from the Luminal subtypes. The third component explains an additional 8% of the variance and differentiates HER2 + samples from most samples with other subtype assignments. Functional enrichment of the selected genes highlighted biological processes related to chromosome segregation, regulation of cell cycle, and programmed cell death (S3 File and S3 Fig in S6 File).

Based on this initial result, two unsupervised clustering methods (k-means and hierarchical) were executed on the counts obtained for the 475 DEGs on the larger set of 204 samples belonging to the 12 studies with at least 9 samples per study. The number of clusters (k parameter) varied from 1 to 10. A weighted jaccard distance was used to evaluate clusters obtained in each experiment, comparing the consistency between methods for the same k value and calculating the agreement with the clustering induced by the predicted subtypes (see methods for details). The clusters obtained from the 475 DEGs were also compared with clusters obtained from the 500 genes with highest variance and the clusters obtained using only the PAM50 genes.

The clusters obtained from the 475 DEGs were more consistent between clustering algorithms across k values, compared to clusters obtained with other sets of genes (Fig 3B). They were also more consistent with the predicted subtypes, compared to clusters obtained from the PAM50 genes (Fig 3C). The best overall consistency (0.43) was achieved running the k-means method on the 475 DEGs with k = 4. This was closely followed by the same method and the same genes with k = 5. Considering that the subtype prediction includes 5 possible subtypes, we selected the latter clustering for further downstream analysis.

### Immune and stromal cell composition and *in silico* estimation of drug sensitivities in clustered transcriptomic profiles

**Fig 4:**
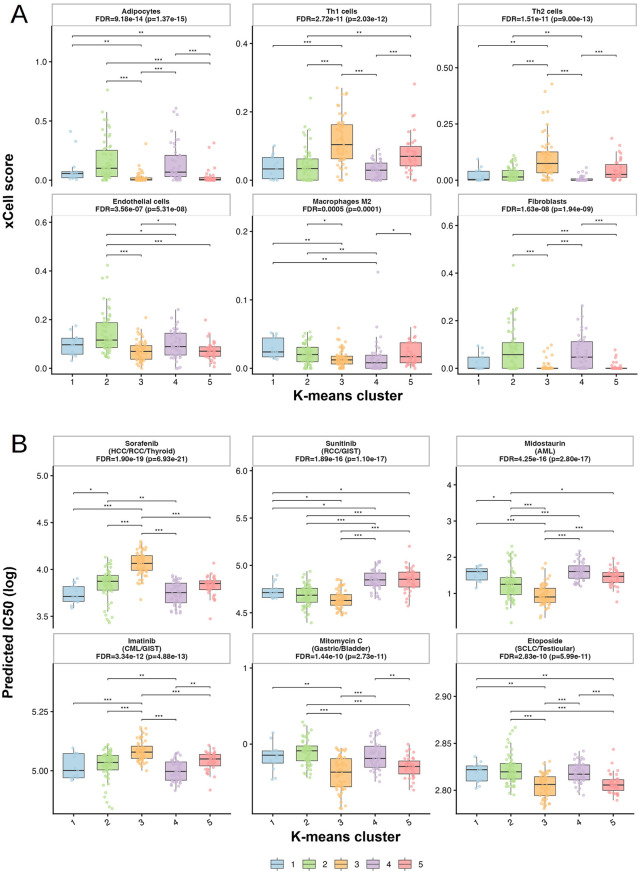
Downstream analysis of clusters of breast cancer samples. (A) Differences in cell subtypes among clusters based on xCell deconvolution analysis. The xCell scores represent the estimated frequencies of cell types within samples. (B) Drug sensitivity predictions, measured as IC50 values, for six FDA-approved drugs across transcriptomic clusters using the pRRophetic R package. Paired comparisons between clusters were performed using the Dunn test, with p-values < 0.05 considered significant. Error bars represent the median and interquartile range.

To explore potential differences in immune cell infiltration across the three identified clusters, we employed the xCell deconvolution method [52], which estimates the frequencies of 67 unique immune and stromal cell types from bulk transcriptomic profiles. From these, 40 subtypes showed significant differences between groups (adjusted p-value <0.01 for a Kruskal Wallis test, S4 File). The cell types with lowest p-values were adipocytes and Th-cells (Fig 4A). Adipocytes were more represented in the clusters 2 and 4, which have most samples subtyped as Normal-like and Luminal A respectively. Conversely, Th1 and Th2 cells were more represented in clusters 3 and 5, which have most samples subtype as Basal and Luminal B respectively. Biologically relevant differences across clusters were observed in Th2 cells, M2 macrophages, and endothelial cells. Th2 cells showed enrichment in cluster 3, suggesting a skewing toward a type 2 immune response in this group. Similarly, M2 macrophages displayed significant variation across clusters, with higher scores in clusters 1 and 5, while cluster 3 consistently showed lower infiltration levels. This pattern may reflect differences in immunosuppressive microenvironmental features among clusters. Endothelial cells were also differentially distributed, with an enrichment in cluster 2 compared to other clusters, indicating potential differences in angiogenic activity across transcriptomic groups. These differences were consistently observed in an analysis of the groups directly inferred from subtype assignments, but with larger p-values overall and lack of significance for the case of macrophages and endothelial cells (S4 Fig in S6 File).

Breast cancer is a highly heterogeneous disease with varying responses to standardized treatments across molecular subtypes. While chemotherapy is effective for many patients, challenges persist in cases of refractory disease and particularly in triple-negative breast cancer (TNBC), where therapeutic options remain limited, and there is a critical need for novel treatments. In this study, we explored potential drug sensitivities through an *in silico* prediction of response to 137 drugs based on the transcriptomic profiles from individual patient clusters. Differential IC50 values were predicted for 113 drugs (adjusted p-value <0.01 for a Kruskal Wallis test, S5 File). Particularly, cluster 3 (mostly composed of samples with basal subtyping) had higher sensitivity for Imatinib and Sorafenib, and lower sensitivity for Etoposide, Midostaurin, Mitomycin C, and Sunitinib, drugs that are already approved to treat other cancers (Fig 4B). The analysis of groups inferred from the predicted subtypes was also consistent in this case, but also with higher p-values and lack of significance of some pairwise comparisons, especially for Etopocide and Mitomycin C (S5 Fig in S6 File).

## Discussion

Given the worldwide importance of breast cancer (BC) as a public health problem, the biology of BC tumors and the development of methodologies for differential identification and diagnosis have been active research topics for more than 25 years [7,53]. In this work, we aimed to provide new knowledge on functional genomics and classification of BC tumors by bioinformatic reanalysis of RNA-seq tumor samples obtained from public databases, originally published by a wide range of studies sampling different populations and having different research goals. The use of current tools for genotyping, machine learning, differential expression, and cell type deconvolution revealed novel information on the estimation of genetic ancestries, clustering and genes with differential expression among patients, leading to predictions of differential treatments.

Although RNA-seq has been available for more than ten years as a tool to obtain a complete characterization of gene expression profiles, clinical diagnosis still relies on immunohistochemistry or on data obtained from chip-based methods targeting a limited number of genes. This is understandable because cost-benefit is an important aspect to consider when making decisions on methods for diagnosis. However, the reductions in sequencing costs and the amount of information that can be obtained in RNA-seq experiments suggest that RNA-seq can become a direct BC subtyping procedure in the near future. Although genotyping based on RNA-seq reads can be subject to biases related to uneven read depth and allele specific expression, recent studies show that global estimates of genetic ancestry could be obtained directly from RNA-seq genotype calls with reasonably good accuracy [54,55]. The results obtained with the panel analyzed in this work support this outcome. This feature enables researchers to obtain genomic variation information in contexts in which it is not feasible or cost-effective to generate DNA sequencing data [56–59]. The analysis was performed taking as reference the hg38 human reference genome due to the amount of annotation resources available for this reference. However, we acknowledge that the inclusion of more complete assemblies such as the T2T [60] or even pangenome graphs including individuals with different ethnicities [61] could improve the accuracy of the analysis. The complete dataset included samples from the five major ancestries described in human population genetic studies. However, the representation disparity among ethnicities was evident in this survey. More than 70% of the samples had European ancestry. This disparity is consistent with those observed in genome-wide association studies in which European ancestry is the most prevalent in public databases [62,63]. Although current large sequencing efforts such as the All of Us sequencing program aim to reduce these disparities for genomic data [64], similar efforts are needed to obtain diverse expression data for cancer samples.

Molecular subtyping following the PAM50 method requires representation of different samples, ideally covering all major subtypes. Hence, predictions can not be made based on isolated samples. Trying to solve this issue by combining local data with publicly available data is problematic because batch effects could be introduced in the analysis. Taking into account this situation, we assessed the robustness of subtype annotations, comparing assignments from corrected counts with assignments based on uncorrected counts of project specific subsets. Although the prediction was consistent for close to 90% of the samples, the probability of assignment was above 0.7 for only 42% of the samples. One of the limitations of the PAM50 model is that it is based on the top 10 genes differentiating each subtype in data obtained from microarray assays [35]. The lack of information across all genes could produce some level of sharing of expression information between subtypes. For example, Luminal A can share expression patterns with Normal-like, which translates into a membership probability of belonging to both subtypes for samples belonging to either subtype. These findings align with recent studies highlighting the limitations of PAM50 in accurately capturing the molecular diversity of breast cancer, particularly in non-European populations [65]. This issue is critical because inaccurate subtype classification can lead to inappropriate treatment regimes, such as prescribing ineffective therapies or overlooking targeted options, ultimately worsening patient outcomes. Alternatively, the experiments of unsupervised clustering from complete expression profiles suggest that the signal present in the RNA-seq profiles could be obscured by a potentially large number of genes with expression patterns not related to the cancer development. Differential expression analysis of samples with consistent subtypes allowed us to propose an initial set of 475 genes which seem to provide a better separation between subtypes, compared to the PAM50 genes. We clarify that the proposed clusters are exploratory and hence they should not be directly used for subtype reassignment without clinical validation. However, the downstream analysis of the obtained clusters suggests that new classification methods could be developed based on complete expression data taking for datasets with better representation of the different subtypes and validated annotations. These datasets should also be more inclusive to achieve a better balance in terms of the ethnicity of the donor patients.

The observed differences between clusters after deconvolution into cell subtypes also suggest that single-cell RNA-seq data can be a great resource to redefine molecular subtypes in breast cancer. The differential enrichment of Th2 cells, M2 macrophages, and endothelial cells across transcriptomic clusters suggests biologically meaningful variation in the tumor microenvironment that is not fully captured by classical subtyping. In particular, clusters enriched in basal-like tumors (cluster 3) showed a distinct immune profile compared to clusters predominantly composed of luminal tumors (clusters 4 and 5), in line with previous studies showing that breast cancer subtypes differ in their immune composition and microenvironmental features [66]. Basal-like tumors have frequently been associated with stronger immune infiltration, although this may coexist with immunosuppressive programs that favor tumor progression and immune evasion. In contrast, luminal tumors are often considered less immune-infiltrated than more aggressive subtypes. Additionally, the enrichment of endothelial cells observed in specific clusters, such as cluster 2 (enriched in normal-like tumors), may reflect differences in angiogenic activity across tumor groups, which is biologically consistent with known subtype-related differences in breast cancer signaling programs [10].

In addition, we identified 113 drugs with significantly different effects across clusters. For instance, 4 drugs approved by the Food and Drug administration (Fig 4B) for other cancers showed the lowest IC50 for cluster 3, composed primarily of basal tumors. Drug repurposing reduces the cost and time to get approval for a new condition, as safety and efficacy have already been tested [67]. This is particularly important for basal tumors, as it is the most aggressive type of cancer and treatment is limited [68]. Our analysis suggests that basal tumors could be sensitive to these medications in diverse ancestry groups. Further validation experiments, including clinical validation are needed to test the safety and efficacy of the drug repurposing predictions obtained from this analysis.

Unsupervised analysis of the currently available expression data grouped tumors in clusters generally consistent with prediction subtypes. The proposed clusters provide improved resolution for downstream analyses such as functional enrichment, immune profiling and drug sensitivity analysis. These analyses revealed insights for new tailored therapeutic approaches, generating hypotheses about personalized treatment strategies for patients with limited therapeutic options. Validation of these insights require the analysis of larger datasets with better balance regarding ethnicity, experimental validation, and clinical studies, to translate them into actionable treatment strategies.

## Methods

### Data collection and initial processing

The Human reference genome hg38 was downloaded from the Broad Institute public database (https://console.cloud.google.com/storage/browser/gcp-public-data--broad-references;tab=objects?prefix=&forceOnObjectsSortingFiltering=false). The complete dataset analyzed in this study consists of publicly available RNA-seq data obtained from breast cancer samples of anonymized patients. The data was recovered from the Sequence Read Archive (SRA) database, using the search term “(breast cancer) AND “Homo sapiens”[orgn:txid9606] NOT cell”, then adding as a filter “fastq” in the File type section, “RNA” in Source section and “Public” in Access section. The results were filtered by a minimum number of 10 million reads. We selected a total of 274 samples based on percentage of mapped reads and the geographical origin of the study. We also included 6 samples of healthy tissue that were used as controls, for a dataset of 280 samples in total. RNA-seq reads were mapped to the human reference genome using HISAT2 (v.2.2.1) [69] obtaining aligned reads in SAM format. SAM files were sorted using Picard (v2.27.4). To assess batch effects, we analyzed the distribution of samples from the same study within the PCA space.

### Ancestry estimation

Regions covered by RNA-seq reads were calculated from BAM files using samtools depth (v.1.16.1) [70] and were joined to generate the set of common regions between all the samples. Genomic variation data from the human reference populations was retrieved from the 1,000 Genomes Project [71] (http://ftp.1000genomes.ebi.ac.uk/vol1/ftp/data_collections/1000G_2504_high_coverage/working/20201028_3202_raw_GT_with_annot). The VCF file was filtered retaining the variants covered by RNA-seq common regions, only women individuals, a minimum MAF value of 0.01, and a minimum number of genotyped samples of 1,850, using the VCFFilter functionality of the NGSEP software [72]. The resulting VCF was merged with that obtained from the breast cancer samples into a single VCF.

The merged VCF file was converted to plink format using vcftools (v.0.1.16) [73], ADMIXTURE input was generated using plink (v1.9) [74]. ADMIXTURE (v.1.3.0) [75] analysis was performed for k values between 1 and 10 with a coefficient of variation (CV) value of 20. We used k = 5 to determine the population based on the major groups sampled in the 1,000 Genomes Project, allowing the model to predict the native american component in latin american admixed individuals. Individuals having the largest membership probability to a single population lower than 0.7 were classified as admixed.

### Extraction of read counts from RNA-seq data and correction of batch effects

Gene counts per gene per sample were calculated with Stringtie (v.1.3.5) [76] from the aligned sequences. A matrix of raw counts was obtained from individual sample files running the script prepDe.py available with the Stringtie distribution. Data for 204 samples belonging to the twelve projects having at least nine samples was selected for downstream analysis. A principal component analysis (PCA) of the uncorrected counts, normalized using the VST method of the DESeq2 R package [77], revealed biases related to the study of origin (S2 Fig in S6 File). Consequently, the raw counts were corrected running CombatSeq [78].

### Molecular subtyping prediction and differential expression analysis

Molecular subtypes were predicted from z-scores calculated from gene count matrices normalized using the VST method. The R package geneFu (v.2.28.0) was used to run the PAM50 algorithm [35,79]. Two separate subtype predictions were performed for each sample, the first using corrected counts from the set of 204 samples, and the second using uncorrected counts from the project of origin of the sample. Moreover five additional predictions were performed including random subsets of 25% of the samples within the project of origin of the sample, ensuring that at least four samples were included in each experiment. The number of times that the predicted subtype agreed with the subtype predicted from the dataset of 204 samples were recorded (S1 File).

The different alternatives of subtype predictions were combined to select a reduced set of samples for differential expression analysis between predicted subtypes. An initial set of 38 of samples was selected with the following criteria: a) Belong to one of the four projects with largest number of samples; b) European ancestry; c) Consistency between project specific subtype and the subtype predicted using the 204 samples; d) Complete agreement of predictions in the subset experiments and assignment probability larger than 0.6 or 80% agreement of predictions in the subset experiments and assignment probability larger than 0.8. Because this initial dataset had no representation of Her2 and Normal-like subtypes and only four samples of Luminal B, a second set of 15 samples was selected for these subtypes relaxing condition d) to allow samples with assignment probability larger than 0.5 and at least 60% of agreement in the experiments with subsets. This increased the representation of the underrepresented subtypes to six, five and eight samples respectively.

Differential expression analysis was performed between samples belonging to each possible pair of subtypes to identify differentially expressed genes (DEGs). The analysis was performed by running the DESeq2 package, which estimates log2 fold changes and calculates adjusted p-values (Benjamini-Hochberg correction) to control for false discovery rates (FDR). Different thresholds of log fold change were selected for each comparison to reduce the overrepresentation of genes differentiating specific pairs of subtypes (S1 Table in S6 File). Genes with absolute log fold change significantly higher than the threshold (adjusted p-value < 0.05) in at least one comparison were added to the set of genes for unsupervised analysis. Functional enrichment analysis of the 475 DEGs was performed using the gProfiler software [80] available at https://biit.cs.ut.ee/gprofiler/gost.

### Unsupervised machine learning of gene counts

Z-scores calculated from corrected counts normalized using the VST method of the DESeq2 were used as input for unsupervised clustering. Different experiments were performed varying the input genes (500 with highest variance, 475 selected DEGs and PAM50 genes) and clustering methods. For each set of genes, a PCA of the z-scores was obtained by running the prcomp function of R. Hierarchical clustering and k-means were executed from the PCA latent space. In both cases the number of clusters varied from 1 to 10. The clusters were compared to each other and to the clustering inferred by the predicted subtypes calculating the jaccard similarity between the cluster assignments. For comparisons against the subtypes a weighted version was implemented in which the weight of each pair of samples is calculated as the average of subtype assignment probabilities. Visualization of PCAs was performed using the ggplot2 package [81].

### *In Silico* drug sensitivity analysis

Drug sensitivity analysis was performed using the R package pRRophetic [82], which predicts the half-maximal inhibitory concentration (IC50) of chemotherapeutic agents based on RNA-seq normalized expression data. Sensitivity differences between the K-means clusters were assessed using the Kruskal-Wallis test, followed by the pairwise Dunn test, All p-values were corrected for multiple testing using the Benjamini-Hochberg (BH) method and we established p > 0.05 as the significance threshold.

### Immune and stromal cell infiltration analysis

Immune and stromal cell infiltration was estimated using the xCell algorithm implemented in R (https://github.com/dviraran/xCell). xCell infers the relative enrichment of 64 immune and stromal cell types based on curated gene expression signatures. Differences in cell-type enrichment across clusters were first assessed using the Kruskal–Wallis test. For cell types showing significant global differences, pairwise comparisons between clusters were performed using Dunn’s test, A significance threshold of adjusted p < 0.05 was applied.

## Supporting information

S1 File Publicly available information for samples analyzed in this study. The file includes predictions of genetic ancestry, subtypes and unsupervised clustering.
(XLSX)

S2 File List of 475 genes selected for unsupervised clustering. (TXT)

S3 File Functional enrichment of 475 genes selected for unsupervised clustering. (CSV)

S4 File Subtype deconvolution analysis of RNA-seq data for the analyzed samples. The file includes the results of samples clustered by the proposed method and clustered by the predicted subtypes.
(XLSX)

S5 File Drug sensitivity predictions from RNA-seq data for the analyzed samples. The file includes the results of samples clustered by the proposed method and clustered by the predicted subtypes.
(XLSX)

S6 File Supplementary tables and figures. (PDF)

## References

[1] Sung H, Ferlay J, Siegel RL, Laversanne M, Soerjomataram I, Jemal A, et al. Global cancer statistics 2020: GLOBOCAN estimates of incidence and mortality worldwide for 36 cancers in 185 countries. CA Cancer J Clin. 2021;71(3):209–49. doi: 10.3322/caac.21660 33538338

[2] Szymiczek A, Lone A, Akbari MR. Molecular intrinsic versus clinical subtyping in breast cancer: a comprehensive review. Clin Genet. 2021;99(5):613–37. doi: 10.1111/cge.13900 33340095

[3] Fragomeni SM, Sciallis A, Jeruss JS. Molecular subtypes and local-regional control of breast cancer. Surg Oncol Clin N Am. 2018;27(1):95–120. doi: 10.1016/j.soc.2017.08.005 29132568 PMC5715810

[4] Joyce DP, Murphy D, Lowery AJ, Curran C, Barry K, Malone C, et al. Prospective comparison of outcome after treatment for triple-negative and non-triple-negative breast cancer. Surgeon. 2017;15(5):272–7. doi: 10.1016/j.surge.2016.10.001 28277293

[5] Ye J, Xia X, Dong W, Hao H, et al. Cellular uptake mechanism and comparative evaluation of antineoplastic effects of paclitaxel-cholesterol lipid emulsion on triple-negative and non-triple-negative breast cancer cell lines. Int J Nanomed. 2016;11:4125–40. doi: 10.2147/IJN.S113638 27601899 PMC5003597

[6] Nedeljković M, Damjanović A. Mechanisms of chemotherapy resistance in triple-negative breast cancer-how we can rise to the challenge. Cells. 2019;8(9):957. doi: 10.3390/cells8090957 31443516 PMC6770896

[7] Perou CM, Sørlie T, Eisen MB, van de Rijn M, Jeffrey SS, Rees CA, et al. Molecular portraits of human breast tumours. Nature. 2000;406(6797):747–52. doi: 10.1038/35021093 10963602

[8] Arpino G, Generali D, Sapino A, Del Matro L, Frassoldati A, de Laurentis M, et al. Gene expression profiling in breast cancer: a clinical perspective. Breast. 2013;22(2):109–20. doi: 10.1016/j.breast.2013.01.016 23462680

[9] Ciriello G, Sinha R, Hoadley KA, Jacobsen AS, Reva B, Perou CM, et al. The molecular diversity of Luminal A breast tumors. Breast Cancer Res Treat. 2013;141(3):409–20. doi: 10.1007/s10549-013-2699-3 24096568 PMC3824397

[10] Eroles P, Bosch A, Pérez-Fidalgo JA, Lluch A. Molecular biology in breast cancer: intrinsic subtypes and signaling pathways. Cancer Treat Rev. 2012;38(6):698–707. doi: 10.1016/j.ctrv.2011.11.005 22178455

[11] Haque R, Ahmed SA, Inzhakova G, Shi J, Avila C, Polikoff J, et al. Impact of breast cancer subtypes and treatment on survival: an analysis spanning two decades. Cancer Epidemiol Biomarkers Prev. 2012;21(10):1848–55. doi: 10.1158/1055-9965.EPI-12-0474 22989461 PMC3467337

[12] Norum JH, Andersen K, Sørlie T. Lessons learned from the intrinsic subtypes of breast cancer in the quest for precision therapy. Br J Surg. 2014;101(8):925–38. doi: 10.1002/bjs.9562 24849143

[13] Goldhirsch A, Wood WC, Coates AS, Gelber RD, Thürlimann B, Senn HJ. Strategies for subtypes--dealing with the diversity of breast cancer: highlights of the St. Gallen International Expert Consensus on the Primary Therapy of Early Breast Cancer 2011. Ann Oncol. 2011;22(8):1736–47. doi: 10.1093/annonc/mdr304 21709140 PMC3144634

[14] Ignatiadis M, Singhal SK, Desmedt C, Haibe-Kains B, Criscitiello C, Andre F, et al. Gene modules and response to neoadjuvant chemotherapy in breast cancer subtypes: a pooled analysis. J Clin Oncol. 2012;30(16):1996–2004. doi: 10.1200/JCO.2011.39.5624 22508827

[15] Ades F, Zardavas D, Bozovic-Spasojevic I. Luminal B breast cancer: molecular characterization, clinical management, and future perspectives. J Clin Oncol. 2014;32(25):2794–803. doi: 10.1200/JCO.2013.54.1870 25049332

[16] Foulkes WD, Stefansson IM, Chappuis PO, Bégin LR, Goffin JR, Wong N, et al. Germline BRCA1 mutations and a basal epithelial phenotype in breast cancer. J Natl Cancer Inst. 2003;95(19):1482–5. doi: 10.1093/jnci/djg050 14519755

[17] Jung J, Kang E, Gwak JM, Seo AN, Park SY, Lee AS, et al. Association between basal-like phenotype and BRCA1/2 germline mutations in Korean breast cancer patients. Curr Oncol. 2016;23(5):298–303. doi: 10.3747/co.23.3054 27803593 PMC5081005

[18] Mavaddat N, Barrowdale D, Andrulis IL, Domchek SM, Eccles D, Nevanlinna H, et al. Pathology of breast and ovarian cancers among BRCA1 and BRCA2 mutation carriers: results from the Consortium of Investigators of Modifiers of BRCA1/2 (CIMBA). Cancer Epidemiol Biomarkers Prev. 2012;21(1):134–47. doi: 10.1158/1055-9965.EPI-11-0775 22144499 PMC3272407

[19] Bertucci F, Finetti P, Birnbaum D. Basal breast cancer: a complex and deadly molecular subtype. Curr Mol Med. 2012;12(1):96–110. doi: 10.2174/156652412798376134 22082486 PMC3343384

[20] Cadoo KA, Traina TA, King TA. Advances in molecular and clinical subtyping of breast cancer and their implications for therapy. Surg Oncol Clin N Am. 2013;22(4):823–40. doi: 10.1016/j.soc.2013.06.006 24012401

[21] Prat A, Pineda E, Adamo B, Galván P, Fernández A, Gaba L, et al. Clinical implications of the intrinsic molecular subtypes of breast cancer. Breast. 2015;24 Suppl 2:S26-35. doi: 10.1016/j.breast.2015.07.008 26253814

[22] Dai X, Li T, Bai Z, Yang Y, Liu X, Zhan J, et al. Breast cancer intrinsic subtype classification, clinical use and future trends. Am J Cancer Res. 2015;5(10):2929–43. 26693050 PMC4656721

[23] Tekesin K, Emin Gunes M, Bayrak S, Akar E, Ozturk T, Altinay S, et al. PTEN loss is a predictive marker for HER2-positive metastatic breast cancer patients treated with trastuzumab-based therapies. J BUON. 2019;24(5):1920–6. 31786856

[24] Zavala VA, Bracci PM, Carethers JM, Carvajal-Carmona L, Coggins NB, Cruz-Correa MR, et al. Cancer health disparities in racial/ethnic minorities in the United States. Br J Cancer. 2021;124(2):315–32. doi: 10.1038/s41416-020-01038-6 32901135 PMC7852513

[25] Marker KM, Zavala VA, Vidaurre T, Lott PC, Vásquez JN, Casavilca-Zambrano S, et al. Human epidermal growth factor receptor 2-positive breast cancer is associated with indigenous American ancestry in Latin American women. Cancer Res. 2020;80(9):1893–901. doi: 10.1158/0008-5472.CAN-19-3659 32245796 PMC7202960

[26] Su Y, Zheng Y, Zheng W, Gu K, Chen Z, Li G, et al. Distinct distribution and prognostic significance of molecular subtypes of breast cancer in Chinese women: a population-based cohort study. BMC Cancer. 2011;11:292. doi: 10.1186/1471-2407-11-292 21749714 PMC3157458

[27] Li E, Guida JL, Tian Y, Sung H, Koka H, Li M, et al. Associations between mammographic density and tumor characteristics in Chinese women with breast cancer. Breast Cancer Res Treat. 2019;177(2):527–36. doi: 10.1007/s10549-019-05325-6 31254158 PMC7304859

[28] Parise CA, Bauer KR, Caggiano V. Variation in breast cancer subtypes with age and race/ethnicity. Crit Rev Oncol Hematol. 2010;76(1):44–52. doi: 10.1016/j.critrevonc.2009.09.002 19800812

[29] Telli ML, Chang ET, Kurian AW, Keegan THM, McClure LA, Lichtensztajn D, et al. Asian ethnicity and breast cancer subtypes: a study from the California Cancer Registry. Breast Cancer Res Treat. 2011;127(2):471–8. doi: 10.1007/s10549-010-1173-8 20957431 PMC4349378

[30] Wisniewski JM, Walker B. Association of simulated patient race/ethnicity with scheduling of primary care appointments. JAMA Netw Open. 2020;3(1):e1920010. doi: 10.1001/jamanetworkopen.2019.20010 31995215 PMC6991290

[31] Roelands J, Mall R, Almeer H, Thomas R, Mohamed MG, Bedri S, et al. Ancestry-associated transcriptomic profiles of breast cancer in patients of African, Arab, and European ancestry. NPJ Breast Cancer. 2021;7(1):10. doi: 10.1038/s41523-021-00215-x 33558495 PMC7870839

[32] Choi J, Jung W-H, Koo JS. Clinicopathologic features of molecular subtypes of triple negative breast cancer based on immunohistochemical markers. Histol Histopathol. 2012;27(11):1481–93. doi: 10.14670/HH-27.1481 23018247

[33] Ochoa S, de Anda-Jáuregui G, Hernández-Lemus E. Multi-omic regulation of the PAM50 gene signature in breast cancer molecular subtypes. Front Oncol. 2020;10:845. doi: 10.3389/fonc.2020.00845 32528899 PMC7259379

[34] Mathews JC, Nadeem S, Levine AJ, Pouryahya M, Deasy JO, Tannenbaum A. Robust and interpretable PAM50 reclassification exhibits survival advantage for myoepithelial and immune phenotypes. NPJ Breast Cancer. 2019;5:30. doi: 10.1038/s41523-019-0124-8 31531391 PMC6733897

[35] Parker JS, Mullins M, Cheang MC, et al. Supervised risk predictor of breast cancer based on intrinsic subtypes. J Clin Oncol. 2009;27(8):1160–7. doi: 10.1200/JCO.2008.18.1370 19204204 PMC2667820

[36] Wallden B, Storhoff J, Nielsen T, Dowidar N, Schaper C, Ferree S, et al. Development and verification of the PAM50-based Prosigna breast cancer gene signature assay. BMC Med Genomics. 2015;8:54. doi: 10.1186/s12920-015-0129-6 26297356 PMC4546262

[37] Veerla S, Hohmann L, Nacer DF, Vallon-Christersson J, Staaf J. Perturbation and stability of PAM50 subtyping in population-based primary invasive breast cancer. NPJ Breast Cancer. 2023;9(1):83. doi: 10.1038/s41523-023-00589-0 37857634 PMC10587090

[38] Gnant M, Filipits M, Greil R, Stoeger H, Rudas M, Bago-Horvath Z, et al. Predicting distant recurrence in receptor-positive breast cancer patients with limited clinicopathological risk: using the PAM50 Risk of Recurrence score in 1478 postmenopausal patients of the ABCSG-8 trial treated with adjuvant endocrine therapy alone. Ann Oncol. 2014;25(2):339–45. doi: 10.1093/annonc/mdt494 24347518

[39] Sestak I, Buus R, Cuzick J, Dubsky P, Kronenwett R, Denkert C, et al. Comparison of the performance of 6 prognostic signatures for estrogen receptor-positive breast cancer: a secondary analysis of a randomized clinical trial. JAMA Oncol. 2018;4(4):545–53. doi: 10.1001/jamaoncol.2017.5524 29450494 PMC5885222

[40] Ross JS, Hatzis C, Symmans WF, Pusztai L, Hortobágyi GN. Commercialized multigene predictors of clinical outcome for breast cancer. Oncologist. 2008;13(5):477–93. doi: 10.1634/theoncologist.2007-0248 18515733

[41] Harris L, Fritsche H, Mennel R, Norton L, Ravdin P, Taube S, et al. American Society of Clinical Oncology 2007 update of recommendations for the use of tumor markers in breast cancer. J Clin Oncol. 2007;25(33):5287–312. doi: 10.1200/JCO.2007.14.2364 17954709

[42] Schaafsma E, Zhang B, Schaafsma M, Tong C-Y, Zhang L, Cheng C. Impact of Oncotype DX testing on ER+ breast cancer treatment and survival in the first decade of use. Breast Cancer Res. 2021;23(1):74. doi: 10.1186/s13058-021-01453-4 34274003 PMC8285794

[43] Shah PD, Nathanson KL. Application of panel-based tests for inherited risk of cancer. Annu Rev Genomics Hum Genet. 2017;18:201–27. doi: 10.1146/annurev-genom-091416-035305 28504904

[44] Troester MA, Sun X, Allott EH. Racial differences in PAM50 subtypes in the Carolina breast cancer study. J Natl Cancer Inst. 2018;110(2):176–82. doi: 10.1093/jnci/djx135 28859290 PMC6059138

[45] Van Alsten SC, Vohra SN, Ivory JM, Hamilton AM, Gao X, Kirk EL, et al. Differences in 21-gene and PAM50 recurrence scores in younger and black women with breast cancer. JCO Precis Oncol. 2024;8:e2400137. doi: 10.1200/PO.24.00137 39013134 PMC11555617

[46] Sweeney C, Bernard PS, Factor RE, Kwan ML, Habel LA, Quesenberry CP Jr, et al. Intrinsic subtypes from PAM50 gene expression assay in a population-based breast cancer cohort: differences by age, race, and tumor characteristics. Cancer Epidemiol Biomarkers Prev. 2014;23(5):714–24. doi: 10.1158/1055-9965.EPI-13-1023 24521995 PMC4011983

[47] Huo D, Hu H, Rhie SK, Gamazon ER, Cherniack AD, Liu J, et al. Comparison of breast cancer molecular features and survival by African and European ancestry in The Cancer Genome Atlas. JAMA Oncol. 2017;3(12):1654–62. doi: 10.1001/jamaoncol.2017.0595 28472234 PMC5671371

[48] Fejerman L, John EM, Huntsman S, Beckman K, Choudhry S, Perez-Stable E, et al. Genetic ancestry and risk of breast cancer among U.S. Latinas. Cancer Res. 2008;68(23):9723–8. doi: 10.1158/0008-5472.CAN-08-2039 19047150 PMC2674787

[49] Jack RH, Møller H, Robson T, Davies EA. Breast cancer screening uptake among women from different ethnic groups in London: a population-based cohort study. BMJ Open. 2014;4(10):e005586. doi: 10.1136/bmjopen-2014-005586 25324320 PMC4202018

[50] Januszewski A, Tanna N, Stebbing J. Ethnic variation in breast cancer incidence and outcomes--the debate continues. Br J Cancer. 2014;110(1):4–6. doi: 10.1038/bjc.2013.775 24398563 PMC3887313

[51] Howlader N, Altekruse SF, Li CI, Chen VW, Clarke CA, Ries LA, et al. US incidence of breast cancer subtypes defined by joint hormone receptor and HER2 status. J Natl Cancer Inst. 2014;106(5):dju055. doi: 10.1093/jnci/dju055 24777111 PMC4580552

[52] Aran D, Hu Z, Butte AJ. xCell: digitally portraying the tissue cellular heterogeneity landscape. Genome Biol. 2017;18(1):220. doi: 10.1186/s13059-017-1349-1 29141660 PMC5688663

[53] Lawton TJ. Update on the use of molecular subtyping in breast cancer. Adv Anat Pathol. 2023;30(6):368–73. doi: 10.1097/PAP.0000000000000416 37746905

[54] Belleau P, Deschênes A, Chambwe N, Tuveson DA, Krasnitz A. Genetic ancestry inference from cancer-derived molecular data across genomic and transcriptomic platforms. Cancer Res. 2023;83(1):49–58. doi: 10.1158/0008-5472.CAN-22-0682 36351074 PMC9811156

[55] Johnson CE, Ran X, Wrobel J, Davidson NR, Greene CS, Epstein MP, et al. An analytic pipeline to obtain reliable genetic ancestry estimates from tumor-derived RNA sequencing data. Cancer Epidemiol Biomarkers Prev. 2025;34(9):1593–9. doi: 10.1158/1055-9965.EPI-25-0371 40622249 PMC12340684

[56] Barral-Arca R, Pardo-Seco J, Bello X, Martinón-Torres F, Salas A. Ancestry patterns inferred from massive RNA-seq data. RNA. 2019;25(7):857–68. doi: 10.1261/rna.070052.118 31010885 PMC6573782

[57] Razi A, Lo CC, Wang S, Leek JT, Hansen KD. Genotype prediction of 336,463 samples from public expression data. bioRxiv [Preprint]. 2024:2023.10.21.562237. doi: 10.1101/2023.10.21.562237 38559266 PMC10979922

[58] Fachrul M, Karkey A, Shakya M, Judd LM, Harshegyi T, Sim KS, et al. Direct inference and control of genetic population structure from RNA sequencing data. Commun Biol. 2023;6(1):804. doi: 10.1038/s42003-023-05171-9 37532769 PMC10397182

[59] Yépez VA, Gusic M, Kopajtich R, Mertes C, Smith NH, Alston CL, et al. Clinical implementation of RNA sequencing for Mendelian disease diagnostics. Genome Med. 2022;14(1):38. doi: 10.1186/s13073-022-01019-9 35379322 PMC8981716

[60] Nurk S, Koren S, Rhie A, Rautiainen M, Bzikadze AV, Mikheenko A, et al. The complete sequence of a human genome. Science. 2022;376(6588):44–53. doi: 10.1126/science.abj6987 35357919 PMC9186530

[61] Liao W-W, Asri M, Ebler J, Doerr D, Haukness M, Hickey G, et al. A draft human pangenome reference. Nature. 2023;617(7960):312–24. doi: 10.1038/s41586-023-05896-x 37165242 PMC10172123

[62] Fitipaldi H, Franks PW. Ethnic, gender and other sociodemographic biases in genome-wide association studies for the most burdensome non-communicable diseases: 2005-2022. Hum Mol Genet. 2023;32(3):520–32. doi: 10.1093/hmg/ddac245 36190496 PMC9851743

[63] Troubat L, Fettahoglu D, Henches L, Aschard H, Julienne H. Multi-trait GWAS for diverse ancestries: mapping the knowledge gap. BMC Genomics. 2024;25(1):375. doi: 10.1186/s12864-024-10293-3 38627641 PMC11022331

[64] The All of Us Research Program Genomics Investigators. Genomic data in the All of Us Research Program. Nature. 2024;627:340–6. doi: 10.1038/s41586-023-06957-x 38374255 PMC10937371

[65] Okimoto LYS, Mendonca-Neto R, Nakamura FG, Nakamura EF, Fenyö D, Silva CT. Few-shot genes selection: subset of PAM50 genes for breast cancer subtypes classification. BMC Bioinform. 2024;25(1):92. doi: 10.1186/s12859-024-05715-8 38429657 PMC10908178

[66] Jiang J, Pan W, Xu Y, Ni C, Xue D, Chen Z, et al. Tumour-infiltrating immune cell-based subtyping and signature gene analysis in breast cancer based on gene expression profiles. J Cancer. 2020;11(6):1568–83. doi: 10.7150/jca.37637 32047563 PMC6995381

[67] Kulkarni VS, Alagarsamy V, Solomon VR, Jose PA, Murugesan S. Drug repurposing: an effective tool in modern drug discovery. Russ J Bioorg Chem. 2023;49(2):157–66. doi: 10.1134/S1068162023020139 36852389 PMC9945820

[68] Obidiro O, Battogtokh G, Akala EO. Triple negative breast cancer treatment options and limitations: future outlook. Pharmaceutics. 2023;15(7):1796. doi: 10.3390/pharmaceutics15071796 37513983 PMC10384267

[69] Kim D, Paggi JM, Park C, Bennett C, Salzberg SL. Graph-based genome alignment and genotyping with HISAT2 and HISAT-genotype. Nat Biotechnol. 2019;37(8):907–15. doi: 10.1038/s41587-019-0201-4 31375807 PMC7605509

[70] Li H, Handsaker B, Wysoker A, Fennell T, et al. The sequence alignment/map format and SAMtools. Bioinformatics. 2009;25(16):2078–9. doi: 10.1093/bioinformatics/btp352 19505943 PMC2723002

[71] The 1000 Genomes Project Consortium. A global reference for human genetic variation. Nature. 2015;526:68–74. 26432245 10.1038/nature15393 PMC4750478

[72] Tello D, Gil J, Loaiza CD, Riascos JJ, Cardozo N, Duitama J. NGSEP3: accurate variant calling across species and sequencing protocols. Bioinformatics. 2019;35(22):4716–23. doi: 10.1093/bioinformatics/btz275 31099384 PMC6853766

[73] Danecek P, Auton A, Abecasis G. The variant call format and VCFtools. Bioinformatics. 2011;27(15):2156–8. doi: 10.1093/bioinformatics/btr330 21653522 PMC3137218

[74] Chang CC, Chow CC, Tellier LC, Vattikuti S, Purcell SM, Lee JJ. Second-generation PLINK: rising to the challenge of larger and richer datasets. Gigascience. 2015;4:7. doi: 10.1186/s13742-015-0047-8 25722852 PMC4342193

[75] Alexander DH, Novembre J, Lange K. Fast model-based estimation of ancestry in unrelated individuals. Genome Res. 2009;19(9):1655–64. doi: 10.1101/gr.094052.109 19648217 PMC2752134

[76] Pertea M, Pertea GM, Antonescu CM, Chang T-C, Mendell JT, Salzberg SL. StringTie enables improved reconstruction of a transcriptome from RNA-seq reads. Nat Biotechnol. 2015;33(3):290–5. doi: 10.1038/nbt.3122 25690850 PMC4643835

[77] Love MI, Huber W, Anders S. Moderated estimation of fold change and dispersion for RNA-seq data with DESeq2. Genome Biol. 2014;15(12):550. doi: 10.1186/s13059-014-0550-8 25516281 PMC4302049

[78] Zhang Y, Parmigiani G, Johnson WE. ComBat-seq: batch effect adjustment for RNA-seq count data. NAR Genom Bioinform. 2020;2(3):lqaa078. doi: 10.1093/nargab/lqaa078 33015620 PMC7518324

[79] Gendoo DMA, Ratanasirigulchai N, Schröder MS, Paré L, Parker JS, Prat A, et al. Genefu: an R/Bioconductor package for computation of gene expression-based signatures in breast cancer. Bioinformatics. 2016;32(7):1097–9. doi: 10.1093/bioinformatics/btv693 26607490 PMC6410906

[80] Kolberg L, Raudvere U, Kuzmin I, Adler P, Vilo J, Peterson H. g:Profiler-interoperable web service for functional enrichment analysis and gene identifier mapping (2023 update). Nucleic Acids Res. 2023;51(W1):W207–12. doi: 10.1093/nar/gkad347 37144459 PMC10320099

[81] Wickham H. ggplot2: Elegant graphics for data analysis. Springer-Verlag New York; 2016. Available from: https://ggplot2.tidyverse.org

[82] Geeleher P, Cox N, Huang RS. pRRophetic: an R package for prediction of clinical chemotherapeutic response from tumor gene expression levels. PLoS One. 2014;9(9):e107468. doi: 10.1371/journal.pone.0107468 25229481 PMC4167990

