# Supplementary material for: Breast cancer clustering integrating complete gene expression profiles and genetic ancestry
Source: PLoS One. 2026 Jul 24;21(7):e0352514. doi: 10.1371/journal.pone.0352514 (PMC13399333; doi:10.1371/journal.pone.0352514)

Supplementary table 1. Differential expression analysis. The bottom triangle displays the minimum log fold change. The upper triangle displays the number of DEGs identified in each comparison.

	Basal	Her2	Luminal A	Luminal B	Normal
Basal		75	83	57	35
Her2	0.5		82	25	90
Luminal A	1.2	0.25		162	33
Luminal B	0.5	0.25	0.25		39
Normal	0.5	0.25	0.25	0.25	

Supplementary Figure 1. Comparison of genotype calls and ancestry estimates obtained from DNA and RNA-seq data for selected admixed samples from the 1000 genomes project. A. Homozygous and heterozygous differences between genotype calls in a total of 9578 SNPs. B. Ancestry estimates based on DNA genotype data (samples HG01113, HG01125, NA19648, NA19654, NA19657) and RNA-seq samples for the same 5 individuals.

A

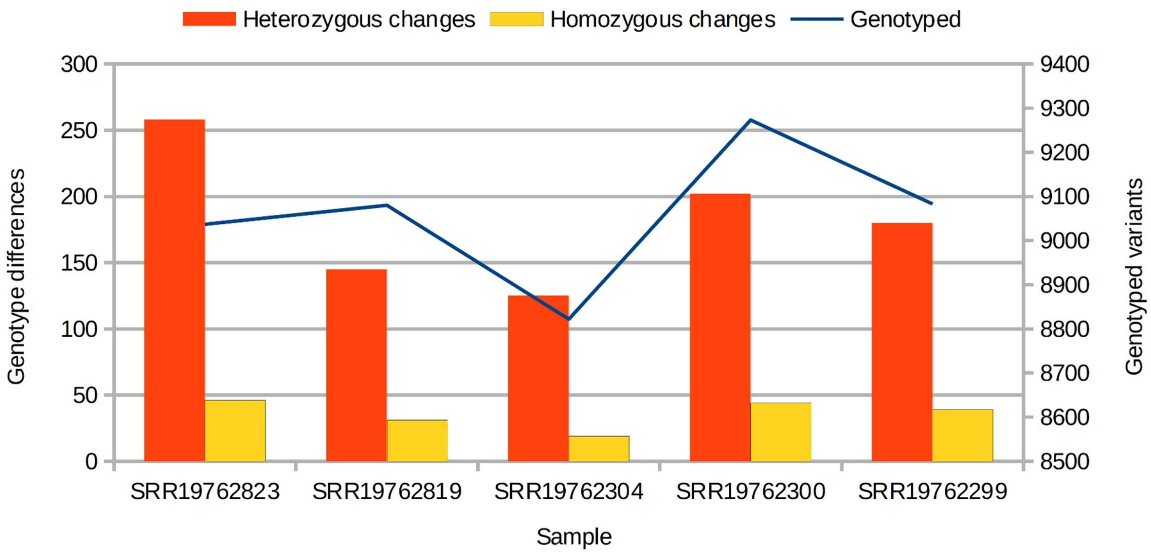

B

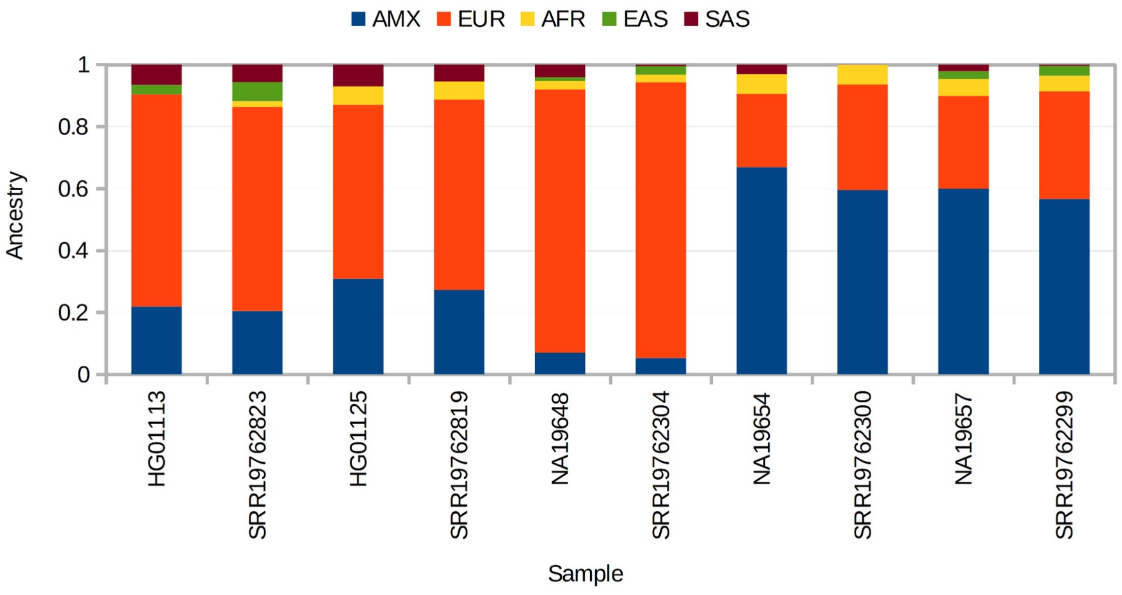

Supplementary figure 2. Principal component analysis of normalized uncorrected counts for 204 samples belonging to the 12 projects with the largest number of samples.

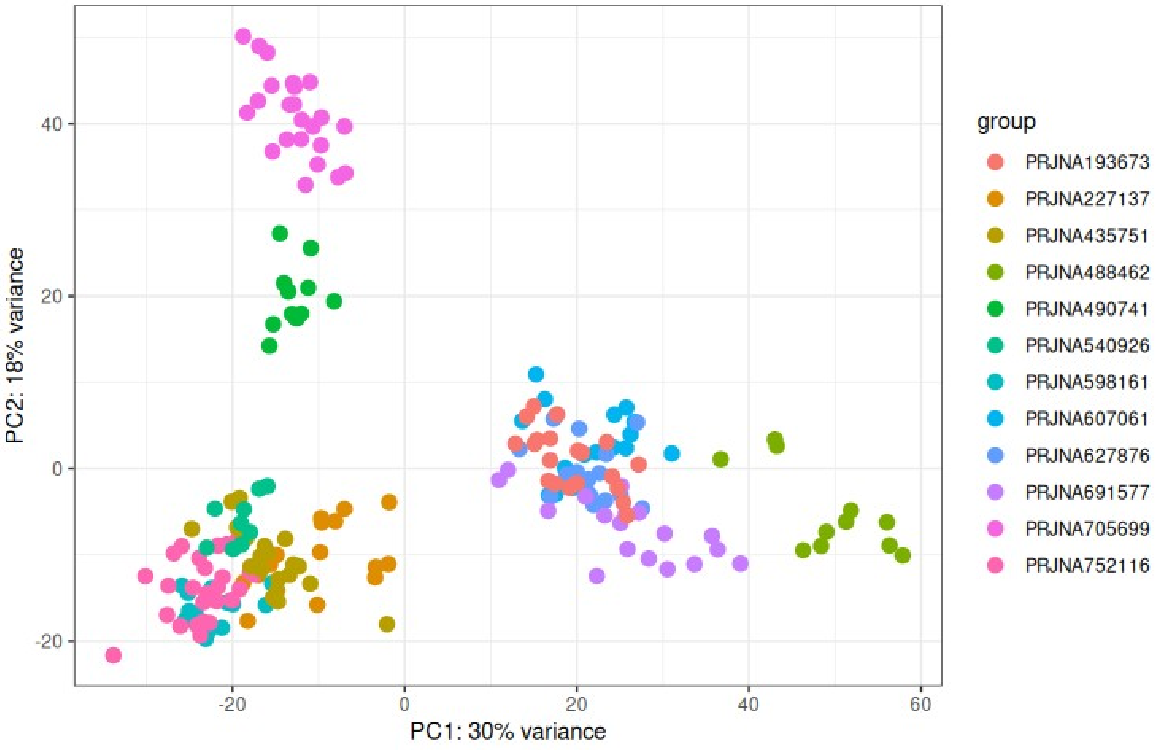

Supplementary figure 3. Enrichment of significant terms for the selected set of 475 genes.

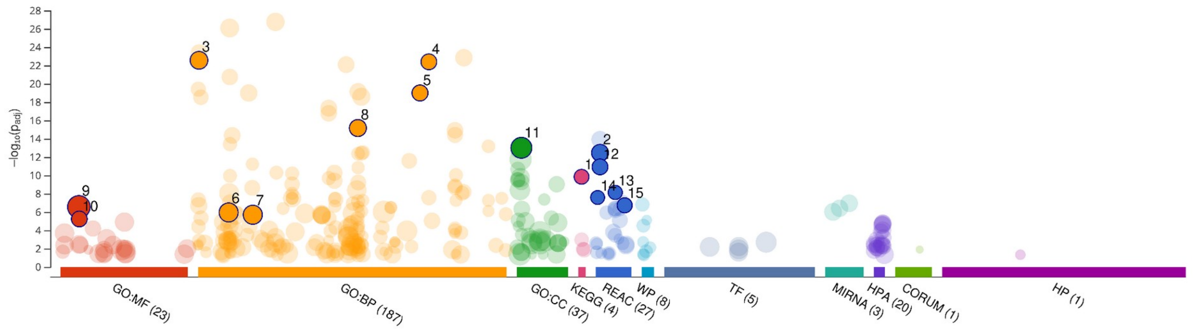

ID	Source	Term ID	Term Name	p_{adj} (query_1)
1	KEGG	KEGG:04110	Cell cycle	1.450×10^{-10}
2	REAC	REAC:R-HSA-69…	Cell Cycle, Mitotic	3.489×10^{-13}
3	GO:BP	GO:0000278	mitotic cell cycle	2.768×10^{-23}
4	GO:BP	GO:0140014	mitotic nuclear division	4.057×10^{-23}
5	GO:BP	GO:0098813	nuclear chromosome segregation	1.017×10^{-19}
6	GO:BP	GO:0006915	apoptotic process	1.147×10^{-6}
7	GO:BP	GO:0012501	programmed cell death	2.036×10^{-6}
8	GO:BP	GO:0051276	chromosome organization	6.864×10^{-16}
9	GO:MF	GO:0005515	protein binding	2.803×10^{-7}
10	GO:MF	GO:0008017	microtubule binding	5.890×10^{-6}
11	GO:CC	GO:0005829	cytosol	9.903×10^{-14}
12	REAC	REAC:R-HSA-69…	Cell Cycle Checkpoints	1.208×10^{-11}
13	REAC	REAC:R-HSA-69…	Mitotic Spindle Checkpoint	7.503×10^{-9}
14	REAC	REAC:R-HSA-14…	Amplification of signal from the kinetochores	2.625×10^{-8}
15	REAC	REAC:R-HSA-24…	Separation of Sister Chromatids	1.893×10^{-7}

**version**	e114_eg62_p19_fa3a7d2c
**date**	4/30/2026, 7:38:21 AM
**organism**	hsapiens

g:Profiler

Supplementary figure 4. Differences in cell subtypes among samples with predicted subtypes based on xCell deconvolution analysis. The xCell scores represent the estimated frequencies of cell types within samples. Paired comparisons between clusters were performed using the Dunn test, with p-values < 0.05 considered significant. Error bars represent the median and interquartile range.

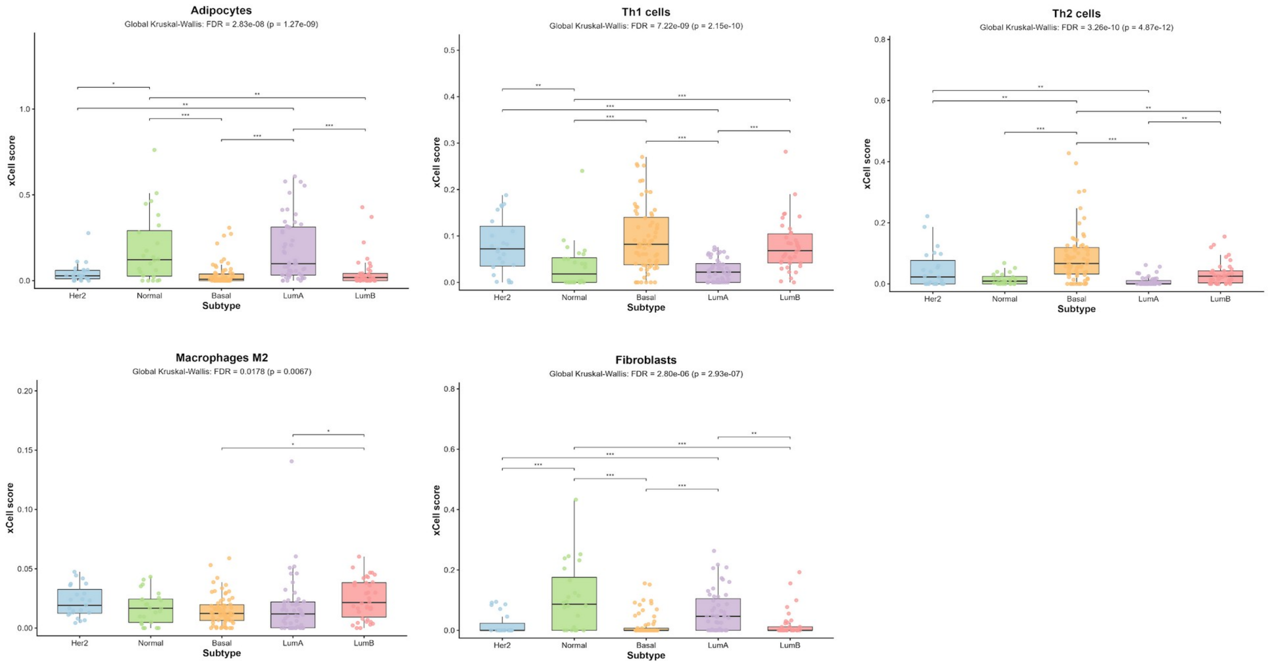

Supplementary figure 5. Drug sensitivity predictions, measured as IC50 values, for six FDA-approved drugs across subtype predictions, using the pRRophetic R package. Paired comparisons between clusters were performed using the Dunn test, with p-values < 0.05 considered significant. Error bars represent the median and interquartile range.

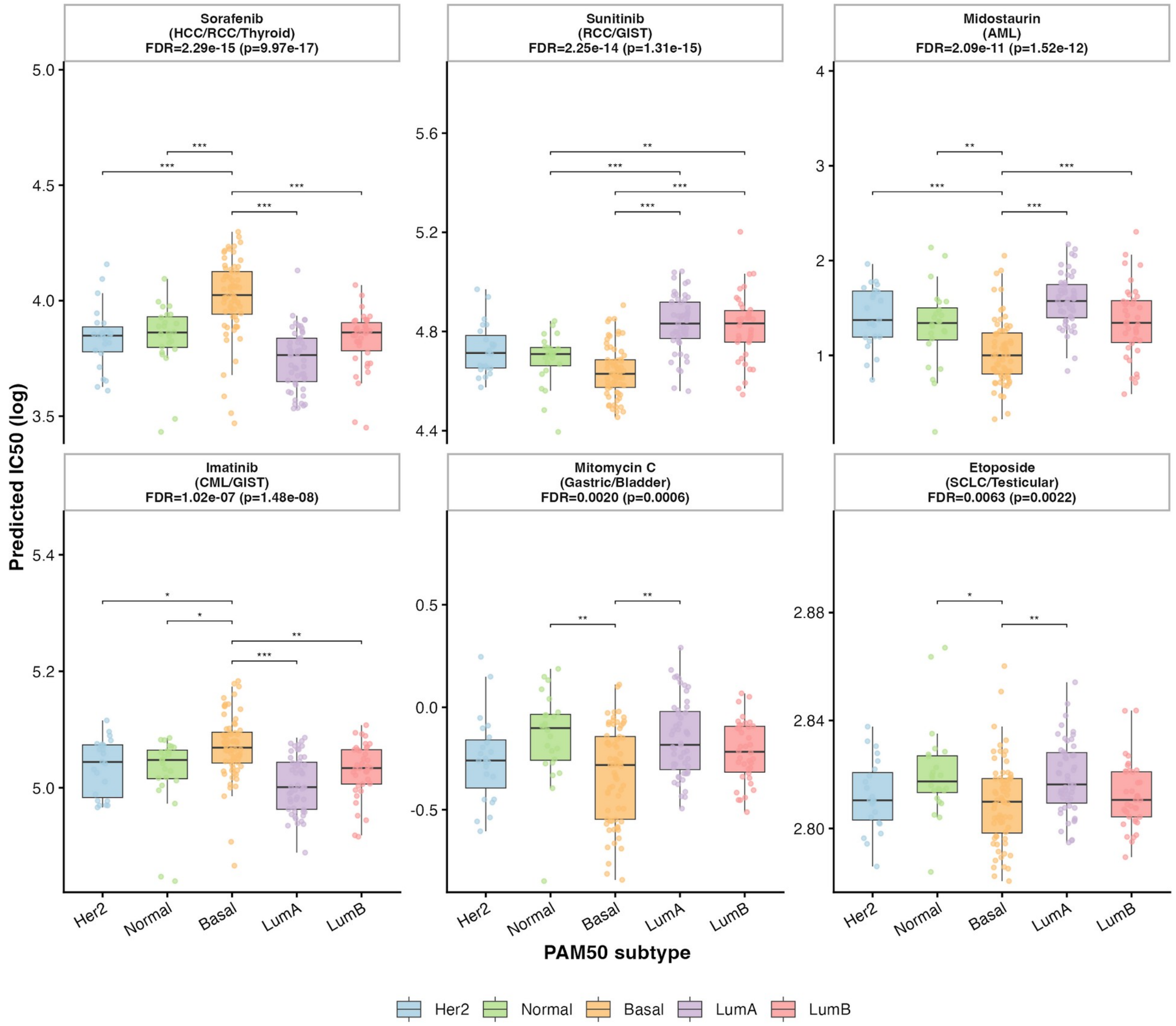

Supplement: S6 File — (PDF) [file pone.0352514.s006.pdf]
